# Antibacterial Evaluation of Synthetic Thiazole Compounds *In Vitro* and *In Vivo* in a Methicillin-Resistant *Staphylococcus aureus* (MRSA) Skin Infection Mouse Model

**DOI:** 10.1371/journal.pone.0142321

**Published:** 2015-11-04

**Authors:** Haroon Mohammad, Mark Cushman, Mohamed N. Seleem

**Affiliations:** 1 Department of Comparative Pathobiology, Purdue University College of Veterinary Medicine, West Lafayette, Indiana, United States of America; 2 Department of Medicinal Chemistry and Molecular Pharmacology, Purdue University College of Pharmacy, and the Purdue Center for Cancer Research, West Lafayette, Indiana, United States of America; Rockefeller University, UNITED STATES

## Abstract

The emergence of community-associated methicillin-resistant *Staphylococcus aureus* (MRSA), including strains resistant to current antibiotics, has contributed to an increase in the number of skin infections reported in humans in recent years. New therapeutic options are needed to counter this public health challenge. The aim of the present study was to examine the potential of thiazole compounds synthesized by our research group to be used topically to treat MRSA skin and wound infections. The broth microdilution method confirmed that the lead thiazole compound and four analogues are capable of inhibiting MRSA growth at concentrations as low as 1.3 μg/mL. Additionally, three compounds exhibited a synergistic relationship when combined with the topical antibiotic mupirocin against MRSA *in vitro* via the checkerboard assay. Thus the thiazole compounds have potential to be used alone or in combination with mupirocin against MRSA. When tested against human keratinocytes, four derivatives of the lead compound demonstrated an improved toxicity profile (were found to be non-toxic up to a concentration of 20 μg/mL). Utilizing a murine skin infection model, we confirmed that the lead compound and three analogues exhibited potent antimicrobial activity *in vivo*, with similar capability as the antibiotic mupirocin, as they reduced the burden of MRSA present in skin wounds by more than 90%. Taken altogether, the present study provides important evidence that these thiazole compounds warrant further investigation for development as novel topical antimicrobials to treat MRSA skin infections.

## Introduction

Ten percent of all hospital admissions in the United States each year are due to patients suffering from skin and soft tissue infections (SSTIs) [1]. SSTIs can range from simple abscesses, cellulitis, and traumatic wound infections to complicated infections (infected burns, diabetic foot ulcers, and major abscesses) [1]. SSTIs are often caused by the bacterial pathogen methicillin-resistant *Staphylococcus aureus* (MRSA) [2, 3]. Indeed, 58% of all SSTIs treated in the United States alone were caused by MRSA, according to an epidemiological study of one national health care system [4]. This agrees with a 2004 study conducted in emergency room departments in 11 cities where MRSA was responsible for 59% of patients presenting with a SSTI [5]. The large number of *S*. *aureus*-based SSTIs has placed a significant economic burden on the healthcare system. A recent report examining the increase in *S*. *aureus*-SSTI hospitalizations in the United States documented a dramatic rise in the annual cost of treating infected patients from $3.36 billion to $4.50 billion (from the years 2001 through 2009) [6]. A recent increase in skin abscesses has been observed and has been associated with a rise in strains of community-associated MRSA (CA-MRSA) [7]. Of these strains, MRSA USA300 has been linked most frequently to skin infections in the United States [5, 8].

According to guidelines provided by the Infectious Diseases Society of America, treatment of moderate to severe skin infections caused by MRSA involves incision and drainage of the affected region combined with administration of empirical antibiotics (such as clindamycin, vancomycin, linezolid, and mupirocin) [9, 10]. However, strains of MRSA exhibiting resistance to several of these antibiotics including vancomycin [11, 12], clindamycin [13, 14], and topical ointments like mupirocin [14–16], indicate that such therapies may be rendered ineffective in the future. Therefore, development of novel antimicrobials capable of treating MRSA-induced SSTIs is an important step necessary to circumvent the burden of this public health issue.

**Fig 1:**
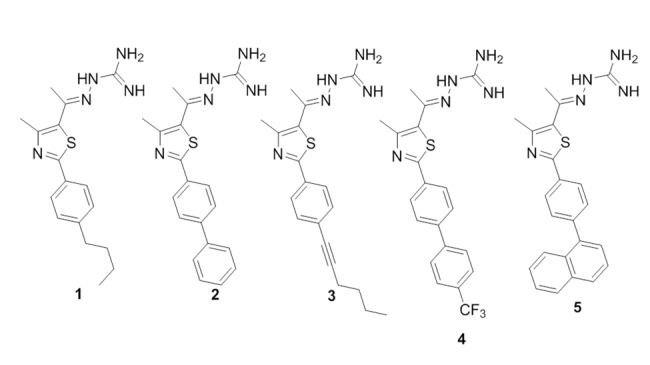
Chemical structures of thiazole compounds 1–5 presented in this study.

Previous research by our group has identified a lead disubstituted phenylthiazole compound (compound **1**, Fig 1) that exhibited potent antimicrobial activity *in vitro* against a diverse array of clinically-significant strains of MRSA [17]. Derivatives of the lead **1** were synthesized to elucidate the structure-activity relationships of this compound. These derivatives revealed that the aminoguanidine moiety at thiazole-C5 is critical for antibacterial activity [18]. Furthermore, a nonpolar, hydrophobic group is favored at thiazole-C2. Analogues to the lipophilic alkyl tail of the lead **1** were subsequently constructed in order to enhance the antimicrobial activity of these thiazole compounds, to improve their toxicity profile, and to refine their physicochemical properties [18]. These particular phenylthiazole compounds possess several excellent characteristics *in vitro* including rapid bactericidal activity against MRSA, a low frequency of bacterial resistance developing, and they have been shown to possess the ability to be used in combination with currently approved antibiotics, such as vancomycin, against MRSA [19]. Studies performed to date indicate these compounds have potential to be used as topical antimicrobials for treatment of MRSA skin infections. The objectives of the current study were to assess the antibacterial activity of the lead thiazole compound and four analogues (Fig 1) *in vitro* against antibiotic-resistant *S*. *aureus* strains isolated from/responsible for skin infections, to assess the ability of these compounds to be paired with mupirocin as a treatment option against MRSA, to confirm the compounds have limited toxicity to human keratinocytes, and to verify the thiazole compounds can retain their antimicrobial activity *in vivo* in an established murine MRSA skin infection model. Confirmation of the ability of these compounds to successfully treat mice infected with a MRSA skin infection will lay the foundation for further assessment of these compounds as novel antimicrobials for treatment of MRSA skin infections.

## Materials and Methods

### Synthesis of thiazole compounds 1–5

Synthetic schemes, spectral data, and purity (>95%, determined by HPLC) of thiazole compounds **1**–**5** (Fig 1), in addition to all intermediates, have been reported elsewhere [17, 18, 20].

### Bacterial strains and reagents used in this study

Clinical isolates of *S*. *aureus* were obtained through the Network of Antimicrobial Resistance in *Staphylococcus aureus* (NARSA) program (Table 1). Clindamycin hydrochloride monohydrate (TCI America, Portland, OR, USA, >98.0% purity) and mupirocin (pure USP) (AppliChem, St. Louis, MO, USA) powders were purchased commercially and dissolved in dimethyl sulfoxide (DMSO) (for clindamycin) or ethanol (for mupirocin) to prepare a stock solution (10 μg/mL). Lipoderm was purchased from the Professional Compounding Centers of America (Houston, TX, USA). Cation-adjusted Mueller-Hinton broth (CAMHB) (Sigma-Aldrich, St. Louis, MO, USA), Tryptic soy broth (TSB) (Becton, Dickinson and Company, Franklin Lakes, NJ, USA), mannitol salt agar (Hardy Diagnostics, Santa Maria, CA, USA), phosphate-buffered saline (PBS) (Sigma-Aldrich, St. Louis, MO, USA), Dulbeco’s modified Eagle’s medium (DMEM) (Sigma-Aldrich, St. Louis, MO, USA), fetal bovine serum (FBS) (USA Scientific, Inc., Orlando, FL, USA), petroleum jelly (Equate [Walmart, Inc.], Bentonville, AR, USA), and 96-well plates (CellTreat Scientific Products, Shirley, MA, USA) were all purchased from commercial vendors.

**Table 1:** Drug-resistant clinical isolates of *Staphylococcus aureus* used in this study.

NARSA^1^ Strain ID	Alternate Strain Designation	US State Isolated From	Year Isolated	Antimicrobial Resistance Phenotype
NRS107	RN4220	Connecticut	1991	Highly mupirocin-resistant and rifampicin
NRS123(MRSA)	USA400	North Dakota	1998	β-lactams
NRS384(MRSA)	USA300-0114	Mississippi	-	Erythromycin, β-lactams, and tetracycline
NRS387(MRSA)	USA800	Washington	-	β-lactams and fluoroquinolones
NRS483(MRSA)	USA1000	Vermont	1993–1994	Erythromycin and methicillin
NRS484(MRSA)	USA1100	Alaska	1996	β-lactams

^1^NARSA, Network on Antimicrobial Resistance in *Staphylococcus aureus*.

### Determination of minimum inhibitory concentration (MIC) against drug-resistant *S*. *aureus* strains

The MIC of thiazole compounds **1**–**5**, clindamycin, and mupirocin was determined against five different MRSA strains and one highly mupirocin-resistant *S*. *aureus* strain isolated from skin wounds, using the broth microdilution method, following the guidelines outlined by the Clinical and Laboratory Standards Institute [21]. A bacterial suspension equivalent to a McFarland standard of 0.5 was prepared and subsequently diluted 1:300 in CAMHB. This bacterial suspension (~1 × 10^5^ colony forming unit (CFU/mL)) was then added to each well of a 96-well microtiter plate. Compounds **1**–**5**, clindamycin, or mupirocin were added (in triplicate) to the first row of the plate and then serially diluted down the ordinate. Plates were incubated at 37°C for 18–20 hours and then the MIC was ascertained. The MIC was classified as the lowest concentration of each test agent where bacterial growth could not be visualized.

### Assessment of synergistic relationship between thiazole compounds and mupirocin against MRSA

The checkerboard assay was utilized to asses if the most potent thiazole compounds (**1**–**3**) have potential to be combined with mupirocin for treatment of MRSA infections [22]. Briefly, a bacterial suspension (1 × 10^5^ CFU/mL) in CAMHB was added to each well of a 96-well microtiter plate. Compounds **1**–**3** and mupirocin were diluted in CAMHB in order to reach the desired starting concentration (2 × or 4 × MIC). Mupirocin was serially diluted along the horizontal axis of the plate while compound **1**, **2**, or **3** was diluted along the vertical axis. Plates were incubated for at least 18 hours at 37°C and the MIC of each compound was recorded. The fractional inhibitory concentration index (ƩFIC) was computed for each combination using the following equation:

$$\sum FIC\;=\;\left(\frac{MICthiazole\;compound\;in\;combination\;with\;mupirocin}{MICthiazole\;compound\;alone}\right)+\left(\frac{MICmupirocin\;in\;combination\;with\;thiazole\;compound}{MICmupirocin\;alone}\right)$$

A FIC index less than or equal to 0.50 was classified as synergism, as described previously [19]. FIC values above 0.50 but less than 4.00 were classified as indifference, while FIC values greater than 4.00 were indicative of antagonism.

### *In vitro* cytotoxicity analysis of thiazole compounds against HaCaT cells

Compounds **1**–**5** were assayed (at concentrations of 5 μg/mL, 10 μg/mL, 20 μg/mL, and 40 μg/mL) against a human keratinocyte (HaCaT) cell line (Catalogue Number: T0020001, AddexBio, San Diego, CA, USA) to determine the potential toxic effect to mammalian skin cells *in vitro* as described before [18]. Briefly, cells were cultured in DMEM supplemented with 10% FBS at 37°C with CO_2_ (5%). Control cells received DMSO alone at a concentration equal to that in drug-treated cell samples. The cells were incubated with the compounds (in triplicate) in a 96-well plate at 37°C with CO_2_ (5%) for two hours prior to addition of the assay reagent MTS 3-(4,5-dimethylthiazol-2-yl)-5-(3-carboxymethoxyphenyl)-2-(4-sulfophenyl)-2*H*-tetrazolium) (Promega, Madison, WI, USA). Absorbance readings (at OD_490_) were taken using a kinetic microplate reader (Molecular Devices, Sunnyvale, CA, USA). The quantity of viable cells after treatment with each compound was expressed as a percentage of the viability of DMSO-treated control cells (average of triplicate wells ± standard deviation). The toxicity data was analyzed via a one-way ANOVA, with post hoc Dunnet’s multiple comparisons test (*P* < 0.05), utilizing GraphPad Prism 6.0 (GraphPad Software, La Jolla, CA).

### *In vivo* assessment of antimicrobial activity of thiazole compounds 1–5 and mupirocin in a MRSA skin infection mouse model

The MRSA murine skin infection study was reviewed, approved, and performed under the guidelines of the Purdue University Animal Care and Use Committee (PACUC) (protocol number: 1207000676) and carried out in strict accordance with the recommendations in the Guide for the Care and Use of Laboratory Animals of the National Institutes of Health. To initiate the formation of a skin wound, eight groups (n = 5) of eight-week old female Balb/c mice (obtained from Harlan Laboratories, Indianapolis, IN, USA) were disinfected with ethanol (70%) and shaved on the middle of the back (approximately a one-inch by one-inch square region around the injection site) one day prior to infection, similar to what has been described elsewhere [23, 24]. To prepare the bacterial inoculum, an aliquot of overnight culture of MRSA USA300 was transferred to fresh TSB and shaken at 37°C until an OD_600_ value of ~1.0 was achieved. The cells were centrifuged, washed once with PBS, re-centrifuged, and then re-suspended in PBS. Mice then received an intradermal injection (20 μL) containing ~2.76 × 10^8^ CFU/mL MRSA USA300. An open wound formed at the site of injection, 48 hours post-infection. Topical treatment was initiated subsequently with each group of mice receiving the following: compound **1**–**5** (2%, using petroleum jelly as the vehicle), mupirocin (2%, using petroleum jelly as the vehicle), compound **1** (2%, using Lipoderm as an alternative vehicle), and a control group receiving the control vehicle (20 mg, petroleum jelly) alone. Each group of mice receiving a particular treatment regimen was housed separately in a ventilated cage with appropriate bedding, food, and water. Mice were checked twice daily during infection and treatment to ensure no adverse reactions were observed. In the event a mouse was observed to become severely ill, the subject was euthanized per the IRB protocol. Mice were treated twice daily for three days. Mice were humanely euthanized via CO_2_ asphyxiation 24 hours after the last dose was administered. The region around the skin wound was lightly swabbed with ethanol (70%) and excised. The tissue was subsequently homogenized in TSB (1 mL). The homogenized tissue was then serially diluted in PBS before plating onto mannitol salt agar plates. The plates were incubated for 20–22 hours at 37°C before viable CFU were counted and MRSA reduction in the skin wound post-treatment was determined for each group. Data were analyzed using a one-way ANOVA, with post hoc Holm-Sidak’s multiple comparisons test (*P* < 0.05), utilizing GraphPad Prism 6.0.

## Results and Discussion

### Antimicrobial activity of thiazole compounds 1–5 against MRSA strains isolated from skin wounds

Previous work has established thiazole compounds **1**–**5** possess potent antimicrobial activity against MRSA (particularly isolates derived from healthcare-associated MRSA cases). To confirm these compounds maintain their antibacterial activity against CA-MRSA strains and MRSA isolates derived from patients presenting with infected wounds (Table 1), the broth microdilution assay was utilized to determine the lowest concentration each compound was able to inhibit the growth of these strains (denoted as the minimum inhibitory concentration or MIC).

When tested against these important clinical isolates of drug-resistant *S*. *aureus*, the thiazole compounds exhibited strong antimicrobial activity similar to (and in several cases better than) mupirocin. As presented in Table 2, the lead thiazole **1** exhibits the most potent activity with a MIC value of 1.3 μg/mL against all six drug-resistant staphylococcal strains tested. The biphenyl and butyne analogues (**2** and **3,** respectively) possess MIC values ranging from 2.8 to 5.6 μg/mL. All five thiazole compounds possess antimicrobial activity against MRSA strains exhibiting resistance to an array of antibiotics including β-lactams, fluoroquinolones (USA800), tetracycline (USA300), and erythromycin (USA300 and USA1000). This indicates cross-resistance between these antibiotics and the thiazole compounds is unlikely to occur. Additionally, the compounds exhibit potent antimicrobial activity against *S*. *aureus* NRS107 (MIC values range from 1.3 to 13.3 μg/mL), a strain exhibiting a high-level of resistance to mupirocin (MIC of 1024.0 μg/mL). Furthermore, compounds **1** and **2** (MIC of 1.3 and 2.8 μg/mL, respectively) are more active than mupirocin (MIC of 4.0 μg/mL) against three additional MRSA strains (USA800, USA1000, and USA1100). Clindamycin, when tested against four of the five MRSA strains, was found to have a MIC of 0.1 μg/mL. This MIC value is similar to what has been reported elsewhere for clindamycin [25]. Collectively, the results confirm that the thiazole compounds do possess potent antimicrobial activity against important CA-MRSA strains and MRSA isolates responsible for infected wounds in patients.

**Table 2:** Minimum inhibitory concentration (MIC in μg/mL) of thiazole compounds 1–5, clindamycin, and mupirocin (tested in triplicate) against five methicillin-resistant *Staphylococcus aureus* (MRSA) and one mupirocin-resistant *S*. *aureus* (NRS107) strain isolated from skin wounds.

	*S*. *aureus* Strain Number					
Compound Number/Name	NRS107	USA300	USA400	USA800	USA1000	USA1100
**1**	1.3	1.3	1.3	1.3	1.3	1.3
**2**	2.8	2.8	2.8	2.8	2.8	2.8
**3**	2.8	5.6	5.6	5.6	2.8	5.6
**4**	13.3	13.3	13.3	13.3	13.3	13.3
**5**	6.4	6.4	12.8	6.4	12.8	6.4
Clindamycin	0.1	1.8	0.1	0.1	0.1	0.1
Mupirocin	1024.0	1.0	1.0	4.0	4.0	4.0

### Combination therapy using thiazole compounds with mupirocin against MRSA

The susceptibility analysis performed with the thiazole compounds indicated they have potential to be used alone for the treatment of MRSA skin/wound infections. While the use of a single agent to treat such infections is often used in the clinical setting, combination therapy using two or more antibiotics is favorable for multiple reasons. Among these reasons include that combination therapy has the potential to slow down the emergence of resistant bacterial strains to antibiotics, to reduce potential negative side effects to patients (by using lower concentrations/doses of each drug), and to alleviate the morbidity related to bacterial infections [26, 27]. Given that multiple topical treatments for skin infections involve a combination of more than one antibiotic, such as Neosporin (consisting of bacitracin, neomycin, and polymyxin B sulfate) and Polysporin ointment (consisting of bacitracin, polymyxin B sulfate, and gramicidin) [28], the identification of compounds to pair with known antibiotics has good potential to expand the available treatment options. Mupirocin has been a key ally in the treatment of MRSA skin infections; however, isolates exhibiting moderate to high-level of resistance to mupirocin (MIC ≥ 512 μg/mL) have emerged, particularly in environments where this antibiotic has been extensively utilized [15, 29, 30]. Identifying agents that can be partnered with mupirocin has the potential to extend the usage of this particular antimicrobial in the clinical setting.

In an earlier study, Alou *et al*, demonstrated that mupirocin forms a synergistic relationship with amoxicillin-clavulanate against MRSA isolates tested *in vitro* via the checkerboard assay [31]. Amoxicllin is a β-lactam antibiotic that interferes with bacterial cell wall synthesis by inhibiting crosslinking of peptidoglycan subunits in the bacterial cell wall [32]. Preliminary studies conducted with our thiazole compounds indicate they also interfere with cell wall synthesis in bacteria; thus we were curious to assess if the thiazole compounds could be used in combination with mupirocin against MRSA, similar to what was found with amoxicillin-clavulanate. Using the checkerboard assay, it was discovered that the most potent thiazole compounds (**1**–**3**) exhibited a strong degree of synergy (FIC index ≤ 0.50) with mupirocin against two of the most prevalent MRSA strains responsible for skin infections (Table 3). Against MRSA USA300, all three compounds exhibited a fractional inhibitory concentration (FIC) index ranging from 0.09 to 0.13 when combined with mupirocin. A similar trend was observed when this combination was tested against MRSA USA400, with FIC values ranging from 0.05 to 0.13. The data provide evidence that supports the prospect that these particular thiazole compounds can be successfully paired with mupirocin to treat MRSA infections (and potentially prolong the utility of mupirocin in the clinical setting).

**Table 3:** Combination testing of thiazole compounds 1–3 with mupirocin against clinically-prevalent strains of community-acquired methicillin-resistant *Staphylococcus aureus* (CA-MRSA).

Compound Number	ƩFIC range^1^MRSA USA300	ƩFIC range MRSA USA400
**1**	0.09–0.13	0.13
**2**	0.09	0.09–0.13
**3**	0.09–0.13	0.05–0.13

^1^ ƩFIC, fractional inhibitory concentration index. Results for the FIC index (ƩFIC) are as follows: ≤ 0.50, synergistic; >0.50 to ≤4.00, indifference; >4.00, antagonistic. ƩFIC range provided is from two independent experiments.

### Toxicity analysis of thiazole compounds to human keratinocytes

**Fig 2:**
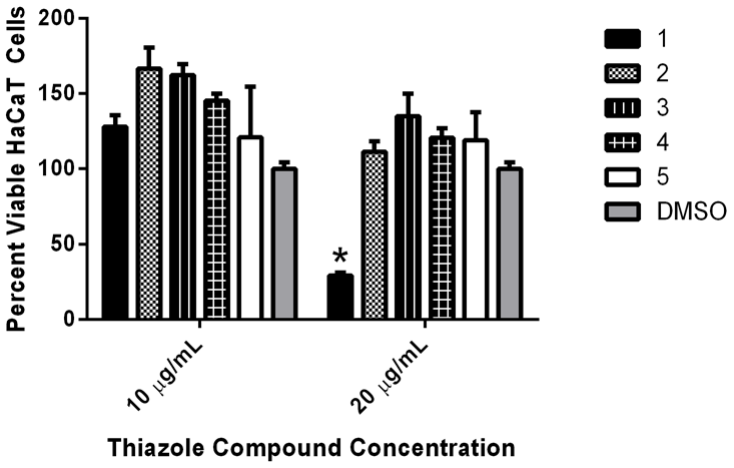
Toxicity analysis of thiazole compounds against human keratinocytes (HaCaT). Percent viable mammalian cells (measured as average absorbance ratio (test agent relative to DMSO)) for cytotoxicity analysis of thiazole compounds **1**, **2**, **3**, **4**, and **5** (tested in triplicate) at 10 and 20 μg/mL against HaCaT cells using the MTS 3-(4,5-dimethylthiazol-2-yl)-5-(3-carboxymethoxyphenyl)-2-(4-sulfophenyl)-2H-tetrazolium) assay. Dimethyl sulfoxide (DMSO) was used as a negative control to determine a baseline measurement for the cytotoxic impact of each compound. The absorbance values represent an average of a minimum of three samples analyzed for each compound. Error bars represent standard deviation values for the absorbance values. A one-way ANOVA, with post hoc Dunnet’s multiple comparisons test, determined statistical difference between the values obtained for compound **1** and DMSO (denoted by the asterisk) (*P* < 0.05).

Selective toxicity is important to ensure compounds with promising antimicrobial activity don’t possess negative side effects to mammalian tissues. Certain regimens (in particular antiseptics) used for treatment of skin infections and wounds have been found to exhibit toxicity to human keratinocytes and impair wound healing, thus limiting their use as therapeutic options [33–36]. Prior to validating the antimicrobial activity of the thiazole compounds in a MRSA skin infection model, it was critical to confirm the thiazole compounds were not toxic to human keratinocytes. Using the MTS assay with a human keratinocyte (HaCaT) cell line, it was confirmed that thiazole compounds **1**–**5** were not toxic at a concentration of 10 μg/mL (Fig 2). Interestingly, the four analogues constructed from compound **1** demonstrated an improved toxicity profile, as they were found to be non-toxic to HaCaT cells up to a concentration of 20 μg/mL. Taken altogether, the data indicate the most potent thiazole compounds *in vitro* (**1**–**3**) are not toxic to human keratinocytes at concentrations up to seven-fold higher than the compounds’ MIC values determined against MRSA.

### Assessment of topical application of thiazole compounds *in vivo* via a murine MRSA skin infection model

As thiazole compounds **1**–**5** exhibited excellent activity against MRSA *in vitro* and displayed no toxicity to human keratinocytes at the compounds’ MIC, we moved to confirm that these compounds could maintain their antimicrobial activity *in vivo*, using an established MRSA murine skin infection model. After the formation of an open wound (infected with MRSA) in the dorsal region of infected mice, each group of mice was treated with a suspension of compounds **1**–**5** (2%), mupirocin (2% suspension), or petroleum jelly (20 mg, used as a vehicle for topical delivery of the compounds/antibiotic) twice daily for three days. The reduction in bacterial burden present in the wounds of infected mice was determined after cessation of treatment. Reduction of bacterial burden in infected wounds is critical to promote proper wound repair and to prevent a severe inflammatory response from being triggered that may negatively impact healing of wounded tissues [37].

**Fig 3:**
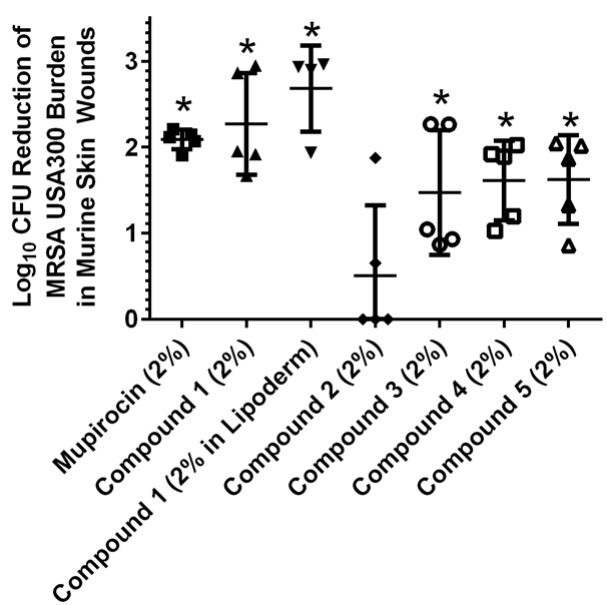
Average log_10_-reduction in MRSA USA300 burden in infected murine skin wounds. Evaluating the effectiveness of treatment of MRSA skin lesions in mice with mupirocin (2%), thiazole compounds **1**–**5** (2%), and compound **1** (2%, using Lipoderm as the vehicle) twice daily for three days. The average log_10_-reduction in bacterial burden (relative to the negative control group (petroleum jelly)) was calculated and presented in the figure. Error bars represent standard deviation values. A one-way ANOVA, with post hoc Holm-Sidak’s multiple comparisons test revealed statistical difference (denoted by asterisk) between compounds **1**, **3**, **4**, **5**, **1** (using Lipoderm as the vehicle), and mupirocin relative to the negative control (*P* < 0.05).

As presented in Fig 3, four thiazole compounds mimic mupirocin’s ability to drastically reduce the burden of MRSA present in skin wounds. Compounds **3**–**5** produce a 1.47 to 1.62 log_10_ reduction in MRSA CFU; this corresponds to a greater than 96% reduction in the bacterial burden, as compared to mice receiving only the vehicle alone (petroleum jelly) for treatment. The lead **1** exceeds the effect of mupirocin, producing a 2.27 log_10_ reduction in MRSA CFU in the skin wound (relative to the 2.07 log_10_ reduction observed with mupirocin). The emergence of increasing resistance to mupirocin, a drug of choice, amongst MRSA strains makes it extremely important to find alternative options for treatment (particularly for skin infections), such as these thiazole compounds. Interestingly, one of the most potent compounds against MRSA USA300 *in vitro* (the biphenyl analogue **2**, MIC of 2.8 μg/mL) is the least effective compound *in vivo* (produces a 0.47 log_10_ reduction in MRSA CFU, that was found to not be statistically significant); this provides a stark reminder that the behavior of compounds *in vitro* needs to be validated with *in vivo* studies to confirm their viability as novel treatment options.

Antimicrobial compounds that can be administered topically (such as thiazole compounds **1**, **3**, **4**, and **5**) for treatment of localized skin lesions have certain advantages over their systemic counterparts. These advantages include the ability to avoid adverse systemic side effects, the ability to localize/concentrate the drug at the target site of infection (providing increased concentration of the drug), lower treatment costs, and a reduced likelihood of inducing bacterial resistance to the treatment agent [36, 38]. Overall, the results garnered from the present study indicate the thiazole compounds (in particular the lead **1**) do warrant further investigation as a topical treatment option for MRSA-infected skin wounds.

### Impact of changing vehicles in reduction of MRSA burden present *in vivo* in infected skin wounds

After confirming four thiazole compounds (**1**, **3**–**5**) have potential for use as novel topical antimicrobials against MRSA, we examined if changing the vehicle used for delivery may further enhance the reduction in bacterial burden present in infected wounds. To assess this, a 2% suspension of the most potent compound (**1**), using Lipoderm as an alternative vehicle, was tested using the murine MRSA skin infection model described above. Lipoderm has been used commercially as a transdermal delivery vehicle to enhance permeation of active pharmaceutical compounds through the skin [39]. It was hypothesized that switching vehicles (from petroleum jelly) to Lipoderm would enhance penetration of the thiazole compounds into the skin wound, thus permitting a greater reduction in the bacterial burden present. As Fig 3 demonstrates, changing vehicles from petroleum jelly to Lipoderm does enhance the reduction in the bacterial load in the skin wound of mice that is achieved by compound **1**. A 0.4-log_10_ improvement in the reduction of MRSA CFU for compound **1** is observed when Lipoderm is used. This corresponds to a > 99.6% reduction in MRSA present in the skin wound after treatment. Thus switching vehicles from petroleum jelly to Lipoderm appears to permit enhanced penetration of the thiazole compounds into skin wounds, leading to an increased reduction in MRSA burden.

## Conclusion

In this study, we demonstrate that five novel synthetic phenylthiazole compounds exhibit potent antimicrobial activity *in vitro* against clinically-relevant strains of MRSA responsible for skin and wound infections. Additionally, compounds **1**–**3** exhibit a strong synergistic relationship when combined with mupirocin against two highly prevalent strains of CA-MRSA. Furthermore, three compounds are not toxic to human keratinocytes at a concentration seven times higher than their MIC against MRSA. The antimicrobial activity of compounds **1**, **3**, **4**, and **5** is confirmed *in vivo* in a murine MRSA skin infection model (> 96% reduction in bacterial load observed, post-treatment). Substitution of the vehicle from petroleum jelly to Lipoderm permits a nearly 0.4-log_10_ additional reduction in bacterial load achieved by compound **1**, indicating this vehicle may be more suitable for enhanced penetration of the compound into infected tissues. Collectively, the results provide valuable information to further develop these thiazole compounds as topical antimicrobial agents for treatment of skin infections and wounds infected by MRSA. Future work with these thiazole compounds includes constructing additional analogues of the lead compound **1** in an effort to improve its potency against MRSA and enhance its toxicity profile with human keratinocytes. Additionally, addressing the limited physicochemical properties of these compounds (through structural modifications of lead compound **1**) is an important next step in order to expand the therapeutic potential of these compounds so they can be administered orally/intravenously for treatment of invasive MRSA infections (both complicated skin infections and systemic infections).
